# A Systematic Review on Clinical Evidence for Topical Metformin: Old Medication With New Application

**DOI:** 10.1002/hsr2.70281

**Published:** 2024-12-19

**Authors:** Kimia Afshar, Sara Adibfard, Mohammad Hossein Nikbakht, Fereshte Rastegarnasab, Mahsa Pourmahdi‐Boroujeni, Bahareh Abtahi‐Naeini

**Affiliations:** ^1^ Student Research Committee Isfahan University of Medical Sciences Isfahan Iran; ^2^ Student Research Committee, School of Dentistry Isfahan University of Medical Sciences Isfahan Iran; ^3^ Skin Diseases and Leishmaniasis Research Center Isfahan University of Medical Sciences Isfahan Iran; ^4^ Pediatric Dermatology Division of Department of Pediatrics, Imam Hossein Children's Hospital Isfahan University of Medical Sciences Isfahan Iran

**Keywords:** dermatology, metformin, periodontitis, topical, wound healing

## Abstract

**Background and Aims:**

Metformin is a widely used oral agent for controlling diabetes mellitus, but it also has other therapeutic benefits for various conditions. In addition, conventional oral metformin, and topical metformin have been used in, in‐vitro studies in the treatment of acne, psoriasis, wound healing, and and so forth. While topical metformin has shown promising results in animal studies, there is limited data on its effectiveness in humans. Our study aims to summarize the clinical findings of human studies on the efficacy of topical metformin.

**Methods:**

This review followed the PRISMA standards and systematically searched multiple databases using specific keywords. The relevant articles were selected according to the inclusions and exclusions criteria.

**Results:**

Our search strategy yielded 1831 articles, after screening, 27 articles met our inclusion criteria which were: human studies, articles published before the start of the search, and topical forms of metformin. We also identified three additional relevant articles through reference checking. Therefore, our systematic review included a total of 30 articles.

**Conclusion:**

Most commonly, topical metformin has been studied in dentistry and dermatology. In dentistry, it has been found effective in treating periodontitis when used with scaling and root planning. Combining metformin with platelet‐rich fibrin can provide better benefits for furcation‐involved teeth, and coating dental implants with metformin improves osseointegration. In dermatology, studies on melasma have had inconsistent results. Topical metformin has also shown promising results in promoting hair regrowth, wound healing, and acne vulgaris, suggesting it could be a potential treatment option for these conditions.

## 1 Introduction

Metformin is a derivative of biguanide that is the most widely used as an oral antihyperglycemic agent for the control of diabetes mellitus (DM) [1]. Oral metformin has been shown to have additional therapeutic benefits for polycystic ovary disease [2], gestational diabetes [3], neurodegenerative diseases [4], coronavirus disease of 2019 (COVID‐19) [4], cardiovascular complications associated with DM [4], reducing cancer risk and improving cancer prognosis [4], and improving fatty liver disease [5]. Also, oral metformin plays an adjuvant role in the treatment of several skin diseases including hormonal acne [6, 7], hidradenitis suppurative (HS) [8], acanthosis nigricans [9], psoriasis [10], and skin cancers [11]. While metformin is widely celebrated for its benefits in managing these conditions, some studies have sparked debate over its potential effects on other diseases [12].

The main mechanism of action for metformin involves enhancing insulin sensitivity, reducing gluconeogenesis, and promoting the absorption of glucose in muscles [13]. Additionally, metformin affects mitochondria, and this results in decreased adenosine triphosphate (ATP) production and rising adenosine diphosphate (ADP) and adenosine monophosphate (AMP) levels, triggering the activation of AMP‐activated protein kinase (AMPK) [1]. AMPK inhibits the transcription of gluconeogenic genes and lipogenesis, thereby improving insulin sensitivity [1].

Metformin has been found to provide significant improvement in various disorders when applied topically. The topical form of metformin has been used in regenerative medicine [14, 15], treatment of acne [16], adjuvant treatment for psoriasis [17], neo‐adjuvant therapy for oral squamous cell carcinoma [18], and delaying or preventing aging‐related cataracts [19]. It has also been investigated for transdermal delivery to enhance wound healing in diabetic patients [20]. Recently metformin has been used as an alternative to hydroquinone for treating pigmentary disorders [21]. Topical metformin reduces the expression of three melanogenic proteins namely tyrosinase, tyrosine‐related protein (TRP)‐1, and TRP‐2. This mechanism has been shown to be effective in reducing hyperpigmentary cutaneous conditions, as demonstrated in an animal experimental study [5].

Although animal and laboratory studies on topical metformin are being conducted and published at an acceptable rate, there have been limited clinical human studies.

In this article, we aimed to categorize the clinical findings of human studies concerning the efficacy of topical metformin on different conditions.

## 2 Materials and Methods

This review was conducted according to the Preferred Reporting Items for Systematic Reviews and Meta‐Analyses (PRISMA) standards. Before commencing the research, all authors thoroughly reviewed the study's inclusion and exclusion criteria and the keywords used to search the literature. The Ethics Committee of Isfahan University of Medical Sciences approved the study (IR.MUI.MED.REC.1402.375).

### 2.1 Literature Search Strategy

A systematic search was performed in PubMed, Scopus, Web of Science, and Embase, until “August 30, 2024,” using the following search strategy limited to the title and abstract, without any filters: (topical) OR (cream) OR (ointment) OR (lotion) OR (gel) OR (paste) AND (metformin). We reviewed and screened the papers based on their titles, abstracts, and full texts. Additionally, we checked related review articles to identify any relevant studies that may have been missed. Furthermore, we examined the references of the selected studies from the full‐text screen to locate any additional articles that may have been overlooked.

### 2.2 Inclusion and Exclusion Criteria

To minimize bias during the selection and extraction of data, first, we established clear inclusion and exclusion criteria to guide the selection process. Studies were selected if they met the following inclusion criteria: (1) human studies, (2) articles published before the start of the search (August 30, 2024), and (3) topical forms of metformin. Narrative and systematic reviews, conference abstracts, in vitro and animal studies, duplicate studies, oral metformin, and adverse effects of metformin and non‐English papers were among the exclusion criteria.

### 2.3 Study Selection and Appraisal

The authors worked collaboratively, discussing their assessments to reach a consensus on the studies included to decrease the selection bias. First, the candidate papers were screened based on their titles by KA, MP, and SA. Subsequently, KA and SA examined the abstracts of the remaining articles, excluding any records that were deemed irrelevant. Finally, KA, FR, and MN attempted to retrieve the full texts of the remaining papers and identified studies that met the inclusion and exclusion criteria for the review. The authors independently completed all processes to minimize the risk of bias. Disagreements between the two researchers were resolved through discussion. If disagreements persisted, a third author (BA) reviewed the study and made the final decision. To enhance the quality of the review, the journal and author names were concealed using a blind method. Full texts of articles that were not open access were obtained upon request from the corresponding author. Duplicate studies were excluded using EndNote software (V.8).

### 2.4 Data Extraction

The extracted data included the following: first author's name, publication year, study design, sample size, mean age, female (*n*) %, treatment protocol, follow‐up period, and main findings. In cases where discrepancies arose between the authors, a third author (BA) was consulted to reach a final agreement (Tables 1 and 2).

**Table 1:** Overview of periodontal disease (randomized clinical trial studies).

First author/year	Sample size; mean age in years (range);	Female (*N*) %	Place of insertion	Treatment protocol	Follow‐up period (months)	Main findings
Sharma et al. [22]/2017	30; 41.4 (25‐50)	16 (54.5%)	Grade II furcation defect	Group 1: OFD + PRF Group 2: OFD + PRF+ metformin 1%	6	Group 2 showed significantly higher probing PD reduction, RVAL, and RHAL gain than Group 1.
Mushtaq et al. [23]/2018	30; NA (NA)	NA	Periodontal pocket	Group 1: SRP Group 2: SRP + metformin 1%	1 and 3	Group 2 showed significantly higher mSBI, PD, and CAL reduction than the Group 1.
Rao et al. [24]/2012	45; 34.6 (30–50)	0 (0%)	Periodontal pocket	Group 1: SRP + metformin 1% Group 2: SRP + placebo	3 and	Group 1 showed significantly higher mSBI, PD reduction, and CAL gain than Group 2. PI decreased in both groups but the difference was not significant.
Pradeep et al. [25]/2015	65; 32.4 (25–50)	27 (41.54%)	Periodontal pocket	Group 1: SRP + metformin 1% Group 2: SRP + placebo	6	Group 1 showed significantly higher IBD, PD reduction, and CAL gain than Group 2. The mSBI was reduced in both groups. This reduction was significantly higher in 3 months but not in 6 months. PI decreased in both groups but the difference was not significant.
Kotry et al. [26]/2016	20; 44.5 (36–55)	9 (45%)	Periodontal pocket	Group 1: SRP Group 2: SRP + metformin 1%	6 m	Group 2 showed significantly higher IBD and PD reduction, and CAL gain and BD increase than Group 1.
Pradeep et al. [27]/2017	64; NA (30–50)	30 (46.88%)	Periodontal pocket	Group 1: SRP + metformin 1% Group 2: SRP + placebo	3, 6, and 9	Group 1 showed significantly higher PI, IBD, mSBI, PD reduction, and CAL gain than Group 2.
Patil et al. [28]/2022	15; NA (25–60)	9 (60%)	Periodontal pocket	Group 1: SRP + placebo Group 2: SRP + metformin 1.5%	3 and 6	Group 2 showed significantly higher PI, IBD, SBI, and PD reduction, and CAL gain than Group 1.
Grace et al. [29]/2017	16; NA (NA)	NA	Periodontal pocket	Group 1: SRP + metformin 1% Group 2: SRP + placebo	1	GI, PD, and CAL decreased in both groups but the differences were not significant.
Arslaan et al. [30]/2022	56; NA (30–50)	NA	Periodontal pocket	Group 1: SRP + metformin 1% Group 2: SRP + alendronate 1%	1.5 and 3	Group 1 showed a significant reduction in PD and CAL compared to Group 2 while PI and mSBI were found nonsignificant.
Mitra et al. [31]/2023	26; 44.5 (34–55)	14 (53.84%)	Intrabony defects	Group 1: SRP + alendronate 1% Group 2: SRP + metformin 1%	3 and 6	Both groups had a significant decrease in PD and gain in CAL. No difference was observed in the efficacy of 1% metformin and 1% alendronate in periodontal therapy of chronic periodontitis patients with infrabony defects.
Mirza et al. [32]/2021	56; NA (30–50)	NA	Periodontal pocket	Group 1: SRP + Doxycycline Group 2: SRP + metformin 1%	1.5 and 3	Group 2 showed a significant reduction in PD and CAL compared to Group 1 while PI and mSBI were found nonsignificant.
Dileep et al. [33]/2018	90; 34.32 (25–45)	46 (51.11%)	Periodontal pocket	Group 1: SRP + placebo Group 2: SRP + rosuvastatin 1.2% Group 3: SRP + metformin 1%	6 and 12	Group 2 and 3 showed a significant reduction in mSBI, PD, and IBD, and higher CAL gain compared to Group 1 while PI was found nonsignificant. IBD reduction was significantly higher in Group 2 than in Group 3.
Kurian et al. [34]/2017	90; 32.54 (24–42)	46 (51.11%)	Periodontal pocket	Group 1: SRP + placebo Group 2: SRP + metformin 1% Group 3: SRP + aloe vera	6 and 12	Groups 2 and 3 showed a significant reduction in mSBI, PI, IBD, and PD, and higher CAL gain compared to Group 1. PD reduction and CAL gain were significantly higher in Group 2 than Group 3.
Swami et al. [35]/2022	21; 43.44 (33–58)	11 (52.38%)	Grade II furcation defect	Group 1: PRF Group 2: PRF + metformin 1%	3, 6, and 12	Group 2 showed significantly higher PD, DV, and HPD reduction, and CAL gain than Group 1.
Pradeep et al. [36]/2015	136; 41 (NA)	68 (50%)	2/3 wall intrabony defects	Group 1: OFD Group 2: OFD + PRF Group 3: OFD + metformin 1% Group 4: OFD + PRF+ metformin 1%	9	Groups 2, 3, and 4 showed significantly higher PD and IBD reduction and RAL and GML gain than Group 1. PD and IBD reduction and RAL gain were higher in Group 4 than in the 2 and 3 groups. PI and mSBI decreased in all groups but the differences were not significant.
Khalifehzadeh et al. [37]/2019	24; 35 (25–45)	15 (62.5%)	Two‐wall intrabony periodontal defects	Group 1: SRP Group 2: SRP + metformin 1% Group 3: SRP + PRGF Group 4: SRP + metformin 1% + PRGF	3 and 6	All the groups exhibited improvements in all the clinical parameters after 6 months. Intergroup comparison of GI, VCAL, and VPD parameters revealed no statistically significant differences. Radiographic changes in Group 4 revealed statistically significant differences compared with other groups; however, there were no statistically significant differences in other groups.
Pradeep et al. [38]/2013	41; 37.2 (30–50)	20 (48.78%)	Periodontal pocket	Group 1: SRP + metformin 0.5% Group 2: SRP + metformin 1% Group 3: SRP + metformin 1.5% Group 4: SRP + placebo	3 and 6	Groups 1, 2, and 3 showed a significant reduction in mSBI, PD, and IBD, and higher CAL gain compared to group 4 while PI was found nonsignificant. Groups 2 and 3 showed greater PD reduction and CAL gain than Group 1. Group 2 showed greater IBD reduction than the other groups.
Sharma et al. [39]/2021	22; 32.3 (18–45)	12 (54.54%)	Implant surface	Group 1: dental implant + metformin 1% Group 2: dental implant	4 and 24	Group 1 showed a significant increase in BD compared to Group 2 while ISQ, OHI‐S, GI, mPI, mSBI, PD, and WKM were found nonsignificant.
Krishnakumar el al. [40]/2023	18; 28.95 (NA)	4 (22.22%)	Implant surface	Group 1: dental implant + metformin 1% Group 2: dental implant	3 and 9	There was no difference between the groups in mPI, mSBI, BD, and crestal bone levels. However, comparing bone volume between the groups at 9 months was statistically significant. Group 1 showed increased bone volume around the implant.
Sobhnamayan et al [41]/2023	26; 9.65 (7–12)	10 (38.46%)	Root canal	Group 1: double antibiotic paste Group 2: double antibiotic paste + metformin 1%	18	The rate of apical closure and root length was significantly higher in Group 2, although the two groups were not significantly different in terms of root width. Canal obliteration was seen in 15% of cases, all of which were in the group 1.sss

Abbreviations: BD, bone density; BOP, bleeding on probing; CAL, clinical attachment level; CAL, clinical attachment loss; DV, defect volume; GI, gingival index; GML, gingival marginal level; HPD, horizontal probing depth; IBD, intrabony defect depth; ISQ, implant stability quotient; mGI, modified gingival index; mPI, modified plaque index; mSBI, modified sulcus bleeding index; *N*, number; NA, not applicable; OFD, open flap debridement; OHI‐S, oral hygiene index‐ simplified; PD, pocket depth; PI, plaque index; PRF, platelet‐rich fibrin; PRGF, plasma rich in growth factor; RHAL, relative horizontal attachment level; RVAL, relative vertical attachment level; SBI, sulcus bleeding index; SRP, scaling and root planing; VCAL, vertical clinical attachment level; VPD, vertical probing depth; WKM, width of keratinized mucosa.

**Table 2:** Overview of melasma, hair regrowth, wound healing, and acne vulgaris studies.

First author/year	Study design	Sample size; mean age in years (range);	Female (*N*) %	Treatment protocol	Main findings
**Melasma**					
Channakeshavaiah et al. [42]/2019	RCT	40; 37.35 (23–84)	32 (80%)	Group 1: 30% metformin lotion, daily for 2 months Group 2: TCC, daily for 2 months	The mean MASI score significantly reduced from the baseline value in both groups, but the reduction was not statistically significant. Topical metformin was found to be safer and better acceptable when compared to TCC.
Mapar et al. [43]/2019	RCT	60; 35.25 (28–42)	NA	Group 1: 15% topical metformin twice daily for 3 months Group 2: placebo twice daily for 3 months	No significant difference was observed between the MASI scores of the patients receiving metformin and the placebo group; however, the MASI Score decrease trend continued until the 12th week; while in the placebo group, no significant decrease was seen after 8 weeks.
AboAlsoud et al. [44]/2021	RCT	40; NA; NA	NA	Group 1: 30% metformin cream daily for 2 months Group 2: TCC daily for 2 months	No significant difference was reported between the two treatment modalities regarding the reduction in melasma throughout the study period.
H M EI‐Komy et al. [45]/2024	A split face, placebo‐controlled	20 (41.35 ± 4.37)	20 (100%)	Metformin side: 30% metformin Placebo side:placebo mask The mask was applied for 4–6 h every week for 12 weeks	At Week 12 and 24, the mean hemi‐MASI score was significantly lower on the metformin side compared to placebo. Metformin has the potential to be an effective topical treatment of melasma with a high safety profile.
Hussain et al. [46]/2024	RCT	62; 41.29 ± 13.01 years and 36.16 ± 11.46	46 (74.19%)	Group 1: 30% Metformin cream and sunscreen for 12 weeks Group 2: 4% Hydroquinone cream, and for 12 weeks	Topical metformin is more effective and safe as compared to 4% hydroquinone in the treatment of epidermal melisma.
**Hair regrowth**					
Araoye et al. [44]/2020	Case report	2; 69, 54; NA	2 (100%)	Case 1: 10% metformin cream 3 times per week and later increased to once daily for 6 months Case 2: 10% metformin once daily for 4 months	Substantial hair regrowth was observed, there might be a potential benefit of topical metformin use in CCCA
Granja et al. [47]/2024	Letter to the Editor	1; 54	1 (100%)	Topical metformin 10% cream once daily + Minoxidil 5% lotion twice daily	Substantial hair regrowth in the vertex region with new repopulated areas and terminal hair follicles after 8 months
**Wound healing**					
Tawfeek et al. [43]/2019	Before‐after open‐label study	30; 32.94 (15–60)	11(36.7%)	Single group: topical metformin hydrogel twice daily for 1 month	Topical metformin hydrogel was found to have a remarkable decrease in pain and edema. The re‐epithelization and formation of new granulation tissue were also observed.
Hedayatyanfard et al. [48]/2022	RCT	53 (35.57 in the treatment group and 36.32 in the case group)	47(88.6%)	Group 1: topical metformin ointment 0.5% twice a day for 3 months Group2: placebo ointment twice a day for 3 months	Metformin topical ointment significantly reduced scar height, vascularity, pigmentation and pliability. No side effects and skin allergies were observed during the study using placebo and metformin ointment.
**Acne vulgaris**					
EL‐Komy et al. [49]/2023	Comparative split‐face study	21; 21.24 ± 1.998 (18–24)	21 (100%)	Right side of face: topical metformin 30% Left side of face: placebo gel	Significant reduction in the counts of comedonal, papular, and nodular lesions was observed on the side treated with metformin compared to the placebo side, at the end of the treatment period with no significant differences in the counts of pustular lesions

Abbreviations: CCCA, central centrifugal cicatricial alopecia; MASI, melasma area and severity index; *N*, number; NA, not applicable; RCT, randomized controlled clinical trials; TCC, triple combination cream.

## 3 Results

### 3.1 Search Result

**Figure 1:**
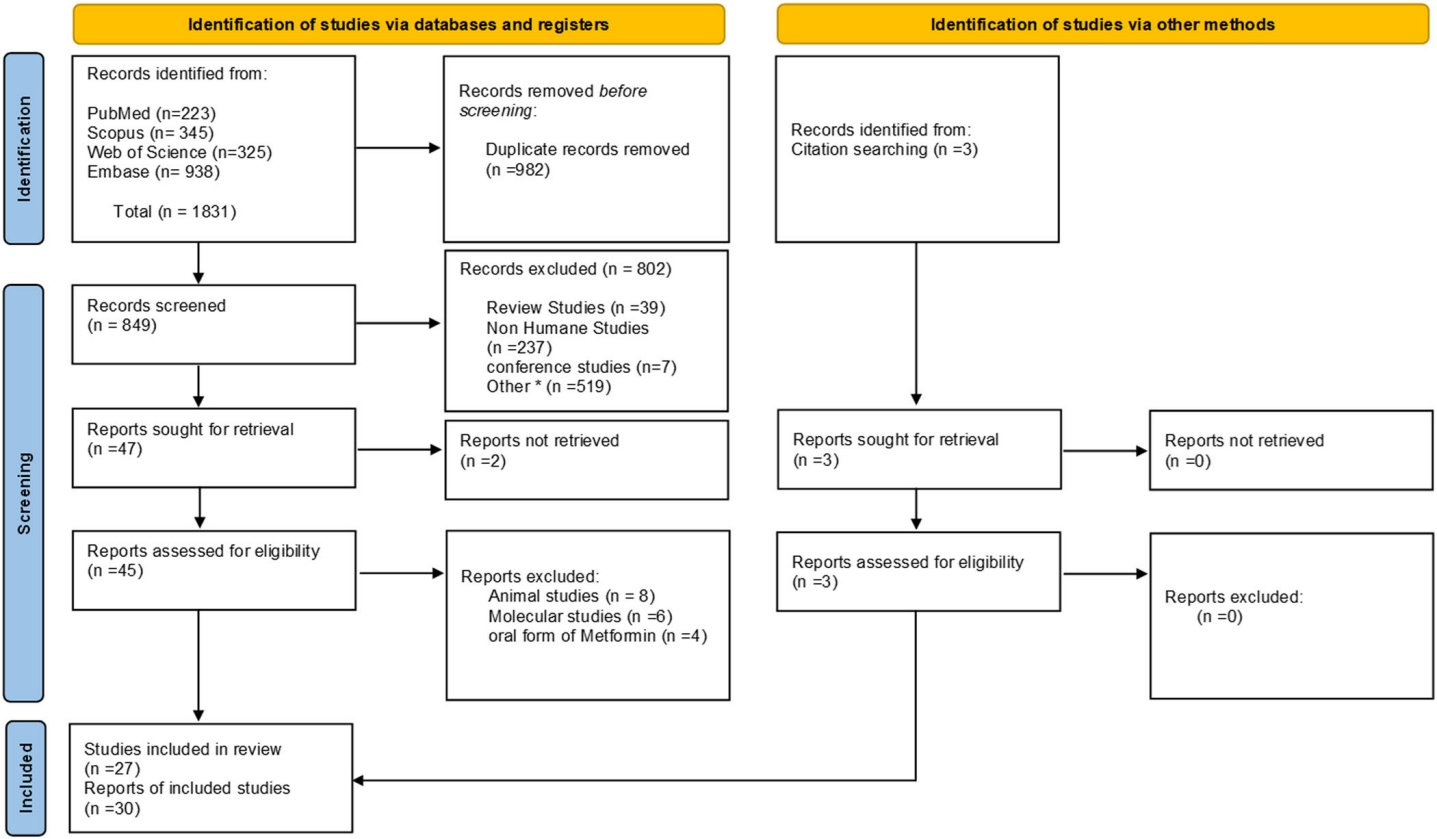
PRISMA flow diagram of the study selection process.

Figure 1 summarizes the process of study selection. Our search strategy identified a total of 1831 articles across PubMed (223), Web of Science (325), Embase (938), and Scopus (345). After using EndNote software (V.8) to remove duplicates, 982 articles were eliminated. The remaining 849 articles underwent screening based on their titles and abstracts, resulting in 47 articles. Unfortunately, the full text of two articles were not found, leaving us with 45 articles for further screening. Eventually, 27 articles met our inclusion criteria. Additionally, we examined the references of these 27 articles and discovered three relevant articles that were not initially identified in our systematic search. In conclusion, our systematic review included a total of 30 articles with 20 focusing on the dental field and discussing periodontal disease and dental implants. The remaining 10 cases are in the dermatologic field and describe the application of topical metformin in four areas: hair regrowth, melasma, acne vulgaris, and wound healing. These studies included 25 RCTs (randomized clinical trial), a case‐control, a letter to editor, a split face, placebo‐controlled, a before‐after open‐label study, and a comparative split‐face study with a total number of 1220 sample size and a range of their age from 7 to 84 years old.

## 4 Discussion

We believe that this review provides valuable insights into the use of topical metformin in both dental and dermatologic settings. It highlights the potential benefits and effectiveness of this treatment approach in improving various conditions.

### 4.1 Chronic Periodontitis

All of the reviewed studies in the treatment of chronic periodontitis were randomized clinical trials that used scaling and root planning (SRP) as a primary treatment (except Sharma et al. [22] study which used open flap debridement) and other materials applied after it (Table 1).

#### 4.1.1 Comparing Metformin With SRP Alone

Mushtaq et al. [23] in 2018 compared using metformin 1% as an adjunct to SRP (test group) with SRP alone (control group) in the treatment of chronic periodontitis in 30 patients. They performed the intervention and assessed the result after one and 3 months. They reported that the percentage of change in modified sulcus bleeding index (mSBI), probing depth (PD), and clinical attachment loss (CAL) was more significant in the test group in 3 months. They concluded that metformin in adjunct with SRP was effective in reducing the clinical parameters (mSBI and PD) and gain in clinical attachment level (CAL*).

#### 4.1.2 Comparing Metformin With Placebo

Six studies compared the application of metformin as an adjunct material to the SRP in the treatment of chronic periodontitis. In the first study of this group which was conducted in 2012 reduction of mSBI, PD, and intrabony defects (IBD) and increase in CAL* was significant between the two groups. However, the reduction of plaque index (PI) was not significantly different between them [24]. Pradeep et al. [25] in 2015 reported a significant reduction in PD and IBD and a significant increase in CAL* in comparison to metformin and placebo groups. In their study, mSBI was significantly decreased in the test group compared to the test group at 3 months but not at the 6‐month follow‐up. Also, their two groups didn't show any significant difference in PI. Kotry et al. [26] in 2016 study showed a significant reduction in PD and IBD and also a significant increase in CAL* and bone density (BD) in the test group compared to the placebo group. Pradeep et al. [27] in 2017 reported a significant decrease in pocket depth (PoD), IBD, and mSBI and a significant increase in CAL* in the test group compared to the placebo group. Also, PI decreased in both groups but the difference was not significant. In the last article of this section in 2022, PI, SBI, probing pocket depth (PD), and IBD were significantly decreased in the test group compared to the control group, and CAL* was significantly increased in that comparison [28]. In contrast with mentioned study, Grace et al. [29] assessed the efficacy of metformin on PD, CAL, and gingival index (GI). They reported that metformin 1% has an effect on the periodontal tissues but it was not significant when compared to a placebo.

In conclusion, among these six studies, five of them suggested that using metformin is useful for the treatment of periodontitis. Grace et al. [29] mentioned less sample size and short‐term follow‐up as the limitations of their study. Maybe their different result depends on these limitations.

#### 4.1.3 Comparing Metformin With Other Substances

Five studies have compared the effect of metformin with alendronate gel, oral doxycycline, rosuvastatin, and aloe vera gel.

Arslaan et al. [30] compared the efficacy of topical metformin and alendronate 1% gel in the treatment of chronic periodontitis. They reported that the metformin group showed a significant reduction in periodontal pocket depth (PD*) and a significant increase in CAL* compared to the alendronate group, while mSBI and PI were found nonsignificant. Mitra et al. [31] designed a study with the same aim and reported that both groups had a significant decrease in PD and gain in CAL. No difference was observed in the therapeutic efficacy of 1% metformin compared to 1% alendronate in the periodontal treatment of chronic periodontitis patients exhibiting infrabony defects.

Mirza et al. [32] compared the efficacy of metformin and systemic doxycycline capsule 200 mg stat, 100 mg OD for 14 days in the treatment of chronic periodontitis. In their study, the metformin group showed a significant reduction in PD* and a significant increase in CAL* compared to the systemic doxycycline group while PI and mSBI were found nonsignificant. Their result was very similar to Arslaan et al. [30]. It seems that the application of metformin gel in conjunction with SRP appears to yield more beneficial outcomes on clinical and biochemical parameters when compared to alendronate gel and systemic doxycycline.

Pankaj et al. [33] compared the efficacy of placebo, Rosuvastatin 1.2% gel, and topical metformin in the treatment of intrabony defects associated with chronic periodontitis. Improvements were observed in PD, mSBI, and CAL* across all treatment groups. Notably, reduction in PD, CAL* gain, and percentage of bone fill were significantly greater in the Rosuvastatin and metformin groups compared to the placebo group. Furthermore, the reduction of IBD was significantly higher in the Rosuvastatin group than in the metformin group in this study.

Kurian et al. [34] conducted a comparative study evaluating the efficacy of placebo, aloe vera gel, and topical metformin in managing IBD associated with chronic periodontitis. Improvements were observed across all treatment groups in GI, bleeding on probing (BOP), PD, and CAL*. Notably, the reductions in mean PD, gains in CAL*, and percentage of bone fill were significantly greater in the metformin and aloe vera groups compared to the placebo group. Furthermore, the application of metformin gel resulted in significantly more favorable outcomes than that of aloe vera gel.

Four studies evaluated the effect of the application of topical metformin along with platelet‐rich fibrin (PRF) and plasma rich in growth factor (PRGF).

Sharma et al. [22] evaluated the clinical and radiographic effectiveness of open flap debridement and PRF when compared to open flap debridement plus PRF and metformin gel in the management of mandibular grade II furcation defects. PRF plus MF group showed significantly higher PD reduction and vertical and horizontal CAL* gain than the PRF alone group. However, mSBI and PI reduction were observed in both groups without significant differences. Swami et al. [35] conducted a study on PRF and PRF + metformin groups. Both the study groups showed improvements in assessed parameters; however, a significantly greater mean reduction of PD, horizontal PD, defect volume, and CAL* gain was observed in the PRF plus MF in comparison with the PRF alone group at 12 months. Pradeep et al. [36] compared the efficacy of open flap debridement (OFD) alone, PRF, metformin gel, and PRF plus metformin gel in the treatment of IBD in chronic periodontitis. metformin gel and PRF plus metformin gel groups showed significant PD reduction and relative attachment level (RAL) gain in comparison with the OFD alone. Between the mentioned groups, a combination of PRF and metformin group showed the best results.

These studies concluded that when PRF is combined with a potential osteogenic agent like metformin, a better therapeutic effect can be achieved for a furcation‐involved tooth.

Khalifehzadeh et al. [37] compared the efficacy of debridement alone as a control group, metformin gel, PRGF, and PRGF plus metformin in the treatment of two‐wall intrabony periodontal defects. All treatment groups showed improvements in all clinical parameters after a 6‐month period. The intergroup analysis indicated that there were no statistically significant differences in the GI, CAL*, and PD among the groups. Although the combination of metformin and PRGF demonstrated statistically significant radiographic changes compared to the control, metformin gel, and PRGF‐only groups, there were no statistically significant differences in other groups. While the application of PRGF combined with metformin effectively enhanced clinical parameters in intrabony two‐wall periodontal defects, these improvements did not differ significantly from those observed in the other groups. In contrast, significant enhancements were noted in the radiographic parameters for the metformin plus PRGF group.

#### 4.1.4 Comparing Metformin in Various Concentrations

Most of the clinical studies in the field of periodontology used metformin 1% gel; However, Pradeep et al. [38] compared the efficacy of placebo and metformin in different concentrations including 0.5%, 1%, and 1.5%. The mean reduction in PD and the mean gain in CAL* were observed to be more pronounced in the metformin treatment groups compared to the placebo group at both the 3‐month and 6‐month assessments. Additionally, a significantly greater decrease in IBD was noted within the metformin groups, with the most substantial reduction occurring in the group treated with 1% metformin. It seems that the 1% metformin plus SRP provides maximum improvement in clinical and radiologic parameters.

### 4.2 Dental Implants Osseointegration

The clinical efficacy of metformin gel in osseointegration was evaluated in two studies [39, 40]. According to the results of the first study in 2021 [39], the mean bone mineral densities in the experimental group exhibited a significant increase when compared to the control group. Additionally, the mean implant stability quotient values for both groups demonstrated a notable increase over the 4‐month period. The result of their study demonstrated coating of implants with metformin gel has a positive bio‐stimulatory effect on osseointegration.

The second study conducted in 2023 [40] indicated no significant differences among the groups concerning the mPI, mSBI, BD, and crestal bone levels. However, a statistically significant difference was observed when comparing bone volume between the groups at the 9‐month follow‐up. Metformin 1% group showed increased bone volume around the implant.

### 4.3 Regeneration of Non‐Vital Immature Teeth

Sobhnamayan et al. [41] conducted a study to examine the impact of combining metformin with double antibiotic paste on the regeneration of non‐vital immature teeth. Their findings indicated that the inclusion of metformin in the double antibiotic paste facilitated root development during the regeneration process. According to the research conducted in the present study, this study is the first study that investigated the effects of metformin in regeneration of the non‐vital immature teeth, and it is suggested to conduct other studies in this field to reach reliable results for practice.

### 4.4 Melasma

Four (RCTs) and one placebo‐control study were conducted to evaluate the efficacy of topical metformin in treating melasma. In all five studies, metformin was compared to a control group that received either a Triple combination cream (TCC), 4% Hydroquinone cream, or a placebo. TCC is a combination of hydroquinone 4%, tretinoin 0.05%, and fluocinolone acetonide 0.01%, and currently is the only approved drug for the topical treatment of melasma. The treatment duration ranged from 8 to 12 weeks, and the main outcome was defined as the change in the melasma area and severity Index (MASI) score [42, 43, 44, 45, 46].

In a study by Channakeshavaiah et al. [42] in 2019, patients with melasma aged over 18 were treated with 30% metformin lotion and TCC. Both groups were followed up every 2 weeks to assess improvement and monitor for adverse effects.

Another study conducted in 2019, involved 60 patients who received 15% topical metformin in Group 1, while the control group received a vehicle for 12 weeks. They were evaluated at Weeks 4, 8, and 12 of the treatment, and 1 month after completion [43].

In 2021, AboAlsoud et al. [44] designed a parallel‐group study comparing 30% metformin and TCC in 40 melasma patients for an 8‐week treatment period.

In 2024, a split‐face, placebo‐controlled study was conducted involving 20 females with melasma. Patients received a 30% loaded peel‐off mask on one side of their face and a placebo mask on the other side, applied weekly for 12 weeks. Hemi‐MASI scores were calculated at baseline, during each visit, and 12 weeks posttreatment [45]

Additionally, Hussain et al. [46] conducted a randomized controlled trial (RCT) in 2024, enrolling 62 patients. Participants in Group A received a 30% Metformin cream along with SPF 30 sunscreen, while Group B applied a 4% Hydroquinone cream and SPF 30 sunscreen, both treatments lasting for 12 weeks. Follow‐ups occurred every 4 weeks during treatment, with a final assessment 1 month after the last session (at the 16‐week mark) [46].

The findings from three studies indicate that while topical metformin may not be significantly more effective than TCC for treating melasma, it appears to have fewer side effects. There is some inconsistency in the results regarding metformin compared to placebo; one RCT found a continuous decrease in MASI scores over 12 weeks with metformin, while a split‐face study suggested that a metformin‐loaded peel‐off mask could be a safe and effective treatment. Notably, metformin showed better efficacy compared to 4% hydroquinone. The inconsistencies can be categorized into three areas: (1) Metformin versus TCC: Two RCTs from 2019 to 2021 with the same sample size (40 participants) and treatment protocol (30% metformin) indicated that metformin is as effective as TCC and safer treatment which is the main treatment for the melasma and also metformin was safer treatment. (2) Metformin versus Placebo: A 2019 study with a stronger design (RCT) and larger sample size (60 vs. 20) found no significant difference between metformin and placebo, whereas another study using a split‐face design observed varying results. The difference in metformin concentration (15% vs. 30%) may also contribute to these discrepancies. (3) Metformin versus Hydroquinone: One study demonstrated significant differences in favor of metformin over 4% hydroquinone.

However, it seems that adding topical metformin into melasma treatment protocol without adding any systemic or cutaneous side effects is a promising option and we can consider topical metformin as a safe and effective treatment for melasma and after more RCTs can be incorporated into current treatment protocols and its potential impact on clinical practice. Also, the percentage of topical metformin and duration of treatment need further research.

In conclusion, further RCTs with larger sample sizes are recommended to assess the efficacy of topical metformin (preferably at 30%) compared to placebo, TCC, and 4% hydroquinone.

The topical application of metformin has been shown to have an anti‐melanogenic effect on reconstituted human epidermis and human skin biopsies [50]. These findings highlight the depigmenting effect of metformin and suggest its potential application in the treatment of hyperpigmentation disorders such as melasma (Table 2).

### 4.5 Hair Regrowth

There is a report of three cases of hair regrowth after the administration of topical metformin for central centrifugal cicatricial alopecia (CCCA). The first case describes a 69‐year‐old black woman with Stage VIa CCCA who experienced hair loss starting in her late 50s. Despite prior treatment with minoxidil, intralesional (IL) triamcinolone acetonide, and topical clobetasol, there was minimal improvement after 5 years. After that, the treatment was switched to topical 10% metformin cream. After 6 months of treatment, substantial regrowth was observed [51]. The second case involves a 54‐year‐old black woman with stage VIa CCCA who noticed hair loss in her early 40s. She received treatment with a combination of IL triamcinolone acetonide, minoxidil, topical ketoconazole, and clobetasol for 9 months with minimal improvement. After 4 months of using the topical 10% metformin cream, notable improvement was observed [51]. The last case was a 54‐year‐old African woman with IIIb phase CCCA refractory to topical minoxidil 5% and topical clobetasol 0.05% who showed substantial hair regrowth in the vertex region with new repopulated areas and terminal hair follicles after 8 months of using topical metformin 10% cream once daily instead of topical clobetasol [47].

These cases highlight the potential benefit of topical metformin in hair regrowing in CCCA. The possible mechanism of metformin in hair regrowing can be related to improving fibrosis in a mouse model via activation of AMPK which reduces the circulating lipids and androgens [51] (Table 2).

### 4.6 Wound Healing

A Before‐after open‐label study was conducted on 30 patients with traumatic ulcer of extremities who were treated with topical metformin hydrogel. The metformin hydrogel was topically applied twice daily on uncovered wounds until the lesions were cleared or for a maximum of 1 month. Histopathological evaluation revealed complete restoration of the connective tissue matrix and re‐epithelization of the wound. It seems that the topical metformin is well‐tolerated and effective option in management of traumatic wounds in healthy individuals [52]. It seems activation of the AMPK pathway, which promotes angiogenesis and rejuvenation is involved in the wound healing effect of metformin [53] (Table 2).

A before‐after open‐label study involved 30 patients with traumatic ulcers on the extremities, treated with topical metformin hydrogel. The hydrogel was applied twice daily to uncovered wounds until complete healing or a maximum duration of 1 month. Histopathological evaluations showed complete restoration of the connective tissue matrix and re‐epithelialization of the wounds. These findings suggest that topical metformin is a well‐tolerated and effective option for managing traumatic wounds in healthy individuals [52]. The wound‐healing effects of metformin may be attributed to the activation of the AMPK pathway, which promotes angiogenesis and tissue rejuvenation [53].

Additionally, a randomized controlled trial (RCT) conducted in 2022 examined the effects of topical metformin on hypertrophic keloid scars. This study included 53 participants, with 28 in the treatment group receiving 0.5% topical metformin ointment and 25 in the control group receiving a placebo. Both groups applied their respective ointments twice daily for 3 months. The Vancouver Scar Scale (VSS) was used to assess improvement. The control group showed no significant changes in mean VSS scores at the 3‐month evaluation, whereas the metformin group exhibited a significant reduction in VSS scores compared to baseline. Notably, no side effects or skin allergies were reported in either group. The results indicate that topical metformin significantly reduces scar height, vascularity, pigmentation, and pliability without adverse effects. This improvement may be linked to metformin's anti‐inflammatory and antifibrogenic properties, suggesting its potential to alleviate hypertrophic and keloid scars [48].

In conclusion, topical metformin hydrogel demonstrates significant promise as an effective treatment for traumatic ulcers and hypertrophic keloid scars. Its ability to promote wound healing and reduce scar formation, coupled with its favorable safety profile, positions it as a valuable addition to current wound management strategies (Table 2).

### 4.7 Acne Vulgaris

Systemic metformin has demonstrated promising results in treating acne. A recent study conducted by EL‐Komy et al. [49] evaluated the safety and efficacy of a 30% topical metformin gel in comparison to a placebo. The study involved 21 female participants who applied metformin and placebo gels to either side of their faces every night for 12 weeks, with follow‐up visits every 4 weeks until week 16. At the end of the treatment period, a significant reduction in the counts of comedonal, papular, and nodular lesions was observed on the side treated with metformin compared to the placebo side. However, by the week 16 follow‐up, there was a notable increase in the mean counts of comedones, papules, and nodules on the metformin‐treated side, indicating a rebound effect posttreatment. In contrast, no significant differences in the counts of pustular lesions were noted between the two sides throughout the treatment period. Importantly, no significant adverse events, including irritation, scaling, erythema, or peeling, were reported by any participants during the study [49]. These findings suggest that topical metformin may serve as a promising and safe therapeutic option for both inflammatory and noninflammatory acne.

#### 4.7.1 Side Effects

Recent studies on melasma treatment have highlighted the safety and efficacy of topical metformin compared to the TCC. In a 2019 study, metformin was well‐tolerated, with only 10% of patients reporting a burning sensation and 5% experiencing both burning and redness from TCC, statistically significant results. Metformin showed no adverse effects on laboratory markers and localized effects, indicating its safety profile. Further evaluations in subsequent studies (2021 and 2024) reinforced these findings. In a 2021 study, no significant adverse events were reported among metformin users, while TCC users experienced mild side effects, including post‐inflammatory hyperpigmentation and irritation, though not statistically significant. By 2024, participants in the metformin group reported no side effects throughout the treatment period, contrasting with TCC users who experienced notable side effects (*p* < 0.001). Overall, these findings suggest that topical metformin is a safer and more tolerable option for treating melasma compared to TCC. However, researchers recommend larger trials for more definitive conclusions.

Also using topical metformin, for the treatment of CCCA, did not exhibit any systemic adverse effects. However, one study noted that patients experienced localized scalp dryness and irritation, which improved with the application of a topical moisturizer or emollient. In the contexts of acne and wound healing, no significant adverse events including irritation, scaling, edema, erythema, or peeling were reported following the administration of topical metformin.

### 4.8 Limitations and Future Direction

Limitations of the Studies on Metformin in Periodontitis are included: Many involved small participant groups, which limits the generalizability of the findings. Additionally, the short follow‐up periods (e.g., 3 months) hinder the assessment of metformin's long‐term efficacy, especially for chronic conditions that may require extended observation. Methodological inconsistencies, such as variations in treatment protocols, metformin concentrations, and evaluation criteria, further complicate the ability to draw definitive conclusions. Differences in control groups (e.g. SRP alone vs. placebo) can introduce bias and affect comparisons. While many studies focused on clinical parameters PD and CAL, they often neglected important factors like patient‐reported outcomes, quality of life, and microbiological assessments. To improve future research, larger sample sizes and multi‐center trials are recommended to enhance statistical power and generalizability. Long‐term follow‐ups are crucial for assessing sustained effects, while standardized treatment protocols and measurement tools will enable more consistent results. It is also essential to control for confounding factors such as patient demographics and systemic health conditions to minimize bias. Future studies should explore the impact of varying metformin concentrations beyond 1% to identify optimal levels for specific conditions. By addressing these limitations, future research can significantly advance our understanding of metformin's role in periodontal therapy, ultimately improving patient outcomes.

Four RCTs and one placebo‐controlled study have been conducted on the treatment of melasma. Due to some inconsistencies in the findings, we recommend further investigation into the optimal concentration of topical metformin and the duration of treatment. Future RCTs with larger sample sizes are essential to evaluate the effectiveness of topical metformin, ideally at a concentration of 30%, compared to placebo, TCC, and 4% hydroquinone within the same study framework.

Currently, there are only a limited number of case reports examining the potential benefits of topical metformin to treat CCCA, so further randomized controlled studies are strongly needed. In the context of wound healing, future research should prioritize optimizing treatment protocols, including timing and formulation, to improve the efficacy of metformin in addressing both acute and chronic scars. Regarding acne treatment, only one recent study was conducted in 2023. Therefore, additional large‐scale RCTs are necessary to confirm the findings of this study and further clarify the long‐term efficacy and safety of topical metformin for acne treatment.

## 5 Conclusion

Topical metformin has been studied in two fields: dentistry and dermatology. In dentistry, it has been found that when used in conjunction with SRP, metformin is effective in treating periodontitis. In the field of dermatology, the results of studies on melasma have been inconsistent. Moreover, topical metformin has shown promising results in promoting hair regrowth, wound healing, and acne vulgaris.

## Author Contributions

**Kimia Afshar:** methodology, writing–original draft, writing–review and editing, investigation, visualization. **Sara Adibfard:** writing–original draft, methodology, investigation, writing–review and editing. **Mohammad Hossein Nikbakht:** writing–original draft, methodology, investigation, writing–review and editing. **Fereshte Rastegarnasab:** writing–review and editing. **Mahsa Pourmahdi‐Boroujeni:** writing–review and editing. **Bahareh Abtahi‐Naeini:** conceptualization, writing–review and editing, methodology, project administration, investigation.

## Conflicts of Interest

The authors declare no conflicts of interest.

## Transparency Statement

The lead author Bahareh Abtahi‐Naeini affirms that this manuscript is an honest, accurate, and transparent account of the study being reported; that no important aspects of the study have been omitted; and that any discrepancies from the study as planned (and, if relevant, registered) have been explained.

## Data Availability

The data that support the findings of this study are available from the corresponding author, upon reasonable request.
